# Complete chloroplast genome sequence and phylogenetic analysis of *Ilex* × *attenuata* ‘Fosteri’ (Aquifoliaceae)

**DOI:** 10.1080/23802359.2021.1970637

**Published:** 2021-08-31

**Authors:** Xinran Chong, Fan Zhang, Yunlong Li, Chuanyong Wang, Yanwei Zhou, Ting Zhou, Yinjie Wang, Xiaoqing Lu, Hong Chen

**Affiliations:** Jiangsu Key Laboratory for the Research and Utilization of Plant Resources, Institute of Botany, Jiangsu Province and Chinese Academy of Sciences, Nanjing, China

**Keywords:** *Ilex* × *attenuata* ‘Fosteri’, complete chloroplast genome, phylogenetic analysis

## Abstract

*Ilex* × *attenuata* ‘Fosteri’ is an important ornamental plant widely distributed in mid-southern China and south-eastern United States. In this study, we assembled the complete chloroplast (cp) genome of *I. attenuata* by high-throughput sequencing and bioinformatics. The full length of cp genome was 157,833 bp with 37.63% overall GC content, which contained two inverted repeats (IR) of 26,093 bp separated by a large single-copy (LSC) and a small single copy (SSC) of 87,188 bp and 18,459 bp, respectively. The cp genome contained 135 genes, including 88 protein-coding genes, 8 rRNA genes and 39 tRNA genes. Phylogenetic tree showed that the close relationship of three species of *Ilex* (*I. attenuata*, *I. viridis* and *I. szechwanensis*) in the Aquifoliaceae family.

*Ilex* × *attenuata* ‘Fosteri’ (Foster, 1940s), also known as Foster’s Holly, is an artificial hybrid *b*etween *I. cassine* and *I. opaca*. Foster’s Holly is a small evergreen tree that is densely pyramidal silhouette. With the characteristics of dark olive-green leaves and persistent bright-red berries, it was planted as an important ornamental plant. However, due to the similar flowers and fruits with other *Ilex* species and cultivars, it is not easy to identify and classify by morphology. With the rapid development of the sequencing technologies, the chloroplast genome has been used to a greater extent in species identification and phylogenetic relationships in plants (Rogalski et al. 2015; Tonti-Filippini et al. 2017). Here, we reported and characterized the complete chloroplast genome of *I. attenuata* in an effort to provide genomic resources useful for promoting its conservation and utilization.

Fresh leaves of *I. attenuata* was collected from Nanjing Botanical Garden, Mem. Sun Yat-sen (E118_83, N32_06), Nanjing, China. The voucher specimen was deposited at the Institute of Botany, Jiangsu Province and Chinese Academy of Science (http://www.cnbg.net/, Hong Chen, chenhong@cnbg.net) under the voucher number NBGJIB-Ilex-0039. Total DNA was extracted using the GMS16011.2.1 Kit (Genmed Scientifics Inc., USA) according to manufacturer’s instructions. After the detection of DNA purity and integrity, high-quality DNA was used to library construction and sequenced using Illumina Noveseq with paired-end 150 strategy. A total of 6357.3 Mb raw data were generated, and 6149.9 Mb clean data were used for the cp genome *de novo* assembly by NOVOPlasty v3.3 (Dierckxsens et al. 2017). Finally, the cp genome annotation was performed by Geseq (Michael et al. 2017) combined with manual correction. The complete cp genome sequence was submitted to Genbank under accession number of MW528026.

The cp genome of *I. attenuata* was a typical quadripartite structure with a length of 157,833 bp, containing two inverted repeat (IR) regions of 26,093 bp, separated by a large single-copy (LSC) and a small single-copy (SSC) region of 87,188 bp and 18,459 bp, respectively. The overall GC content of the cp genome was 37.63%. A total of 135 genes were predicted, including 88 protein-coding genes, 8 rRNA genes and 39 tRNA genes. Eight protein-coding genes, four rRNA genes and seven tRNA genes are duplicated in IR regions. Besides, fifteen genes contained two exons and three genes (*clpP*, *ycf3* and *rps12*) contained three exons.

To explore the phylogenetic position and evolutionary relationship of *I. attenuata*, we selected 14 *Ilex* species and one *Helwingia himalaica* as outgroup for the phylogenetic analysis (Yao et al. 2016; Cascales et al. 2017; Park et al. 2019; Su et al. 2020). The phylogeny was constructed by maximum likelihood (ML) method based on 78 common protein-coding genes extracted from the complete cp genomes of 16 species using PhyML version 3.0 software (Liu et al. 2019). Bootstrap values were estimated from 1000 replicates. In Figure 1, the phylogenetic tree revealed the presence of four clades within *Ilex*, in agreement with the fossil record (Yao et al. 2021), and also fit well with recent reports on plastid phylogenetic analysis (Yao et al. 2016; Su et al. 2020). *I. attenuata* was clustered with *I. viridis* and *I. szechwanensis* in section *Paltoria* in clade III, indicating that it has a relatively close relationship with the two *Ilex* species. The cp genome sequence of *I. attenuata* will provide a useful resource for the conservation genetics of this species as well as for building the phylogenetic relationships of Aquifoliaceae.

**Figure 1.**
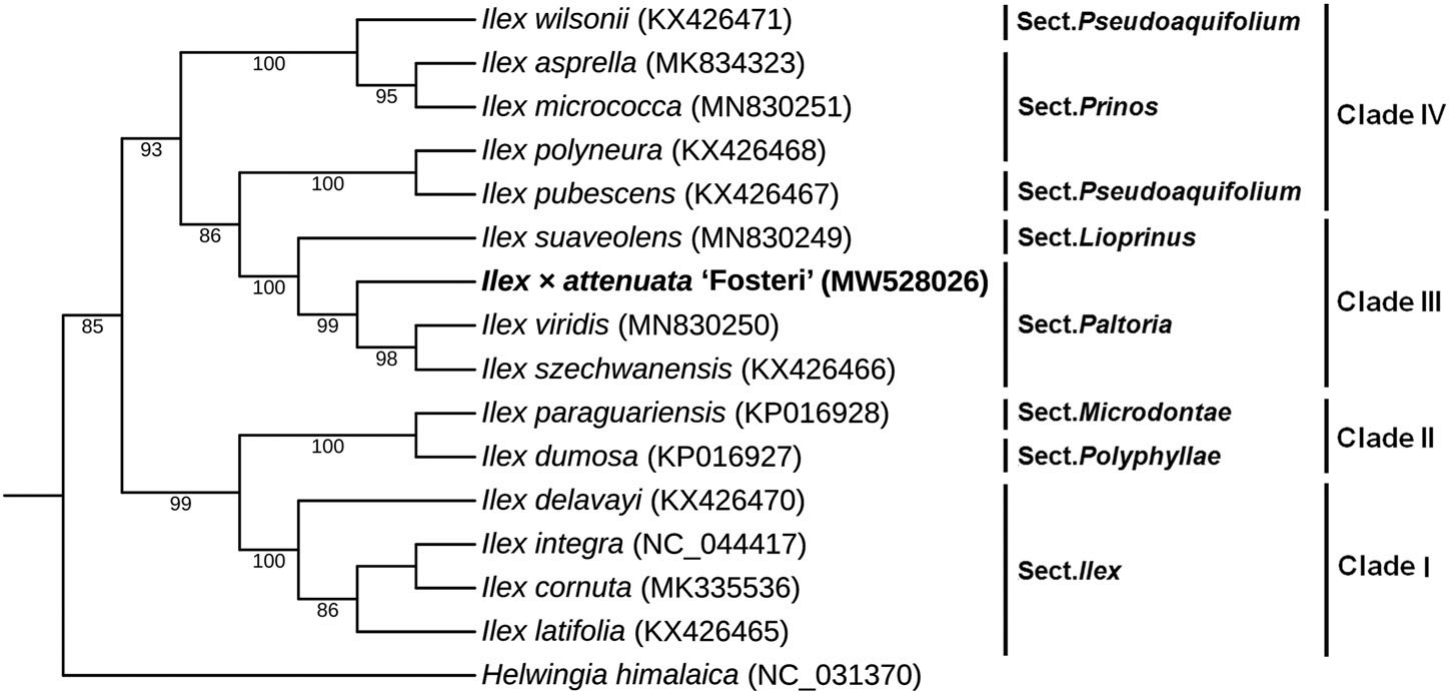
ML phylogenetic tree of *I. attenuata* with 15 species was constructed based on 78 protein-coding genes. Section names were displayed in the right side of phylogenetic tree. Numbers on the nodes were bootstrap values from 1000 replicates. *Helwingia himalaica* was selected as outgroup.

## Data Availability

The genome sequence data that support the findings of this study are openly available in GenBank of NCBI at (https://www.ncbi.nlm.nih.gov/) under the accession no. MW528026. The associated BioProject, SRA, and Bio-Sample numbers are PRJNA690233, SRR13376222, and SAMN17248681, respectively.
